# Hematological Profile of Patients Having Malaria-positive Peripheral Blood Smears: A Cross-sectional Study at a Diagnostic Research Center in Khyber Pakhtunkhwa, Pakistan

**DOI:** 10.7759/cureus.3376

**Published:** 2018-09-27

**Authors:** Inam Ullah, Muhammad U Ali, Saeed Ali, Ahmad Rafiq, Zeeshan Sattar, Sana Hussain

**Affiliations:** 1 Pathology, Khyber Medical College, Peshawar, PAK; 2 General Surgery, Royal Lancaster Infirmary, Lancaster, GBR; 3 Internal Medicine, Florida Hospital, Orlando, USA; 4 Internal Medicine, Khyber Teaching Hospital, Peshawar, PAK

**Keywords:** malaria, thrombocytopenia, p. falciparum, p. vivax, leukopenia, anemia, khyber pakhtunkhwa

## Abstract

Malaria is a life-threatening infectious disease that, in severe cases, is associated with calamitous complications and far-reaching consequences within a community. It is usually manifested by abnormalities in various hematological indices with anemia and thrombocytopenia being the most frequent ones. The present study sheds light on the laboratory profile of patients suffering from malaria and provides a comprehensive analysis and correlation with the available literature worldwide. The study was carried out as a cross-sectional study at OK Diagnostic Lab and Research Center in Peshawar from October 2010 to October 2013. All malaria parasite (MP)-positive cases reported at OK Lab during the study period were employed in the study, making a total of 136 MP positive cases. Complete blood pictures with platelet counts were obtained in all patients and various hematological indices were analyzed according to the World Health Organization (WHO) criteria. Thrombocytopenia was defined as a platelet count of < 150 × 10^3^/cmm and anemia as an hemoglobin (Hb) < 13 g/dL in males and < 12 g/dL in females. Among the 136 MP positive patients, 74 (55.4%) had associated thrombocytopenia while 105 (77.2%) patients showed anemia on a peripheral blood smear. This was followed by leukopenia in 8.8% of cases. Among patients with *Plasmodium falciparum* (*P. falciparum*) infection, anemia was present in 80% of cases as compared to 74% cases with *P. vivax* infection (p = 0.5). Thrombocytopenia was associated with *P. vivax* infection in 71.4% of cases in contrast to P. falciparum infection, where 26% of cases had associated thrombocytopenia (p = 0.01). On the contrary, leukopenia was more prevalent in *P. falciparum* patients (18%), followed by *P. vivax* (2.6%), and mixed parasitemia (11.1%) (p < 0.001). In addition, the study showed statistically significant variations in hematocrit (Hct), mean corpuscular volume (MCV), mean corpuscular hemoglobin concentration (MCHC), and platelet counts across different malarial species (p < 0.05). Likewise, variations within mean Hct levels among males and females were statistically significant, with females showing lower mean Hct levels than males (p < 0.05).

## Introduction

Malaria is considered to be a life-threatening infectious disease that, in severe cases, is associated with calamitous complications and can inflict drastic and far-reaching consequences within a community. The existence of this disease can be traced back to 2700 years BC in China and historians have even reported Alexander to be a victim of it during the battle of Mesopotamia in the fourth century BC [1]. The disease is caused by infection with a parasitic unicellular organism of genus *Plasmodium*, which gets injected into the human bloodstream through the bite of a female Anopheles mosquito [2]. Traditionally four species of *Plasmodium, *including *P. falciparum*, *P. vivax*, *P. ovale*, and *P. malariae,* have been known to cause infections in humans. However, another species, *P. knowlesi* which causes malaria in macaque monkeys [3], has been reported to cause malaria in humans [4]. Since 2004, increasing data has been published with regards to an increase in its incidence in various Southeast Asian countries [5].

According to the World Health Organization (WHO) estimates, 40% of the world's population is at risk of developing malaria [1]. Studies have reported a global incidence of 300 - 500 million cases per year with an associated two million deaths per annum [1]. Likewise, in Pakistan, the disease plays havoc with lives of millions per year and local literature reveals higher mortality rates among infants, children, and pregnant women. The National Malaria Control Program of Pakistan has reported a six-fold increase in the incidence of *P. falciparum* malaria that now comprises 42% of all malaria cases reported in the country [6]. Therefore, in addition to being a major public health issue, the disease significantly adds to the country’s economic burden.

In addition to the typical features, including high-grade fever, shivering, vomiting, and jaundice, malaria is frequently associated with hemolytic anemia, hemoglobinuria, and varying degrees of thrombocytopenia [1, 7], with cerebral malaria and renal failure being the most dreaded complications. Although various studies have reported thrombocytopenia in association with malaria as a common finding [1, 8], its correlation with the type of malaria and various hematological parameters has not been evaluated extensively in large studies. In view of the paucity of data from Pakistan, we have attempted to throw some light on the laboratory profile of patients suffering from malaria by analyzing and correlating various hematological indices and comparing them with the available literature worldwide.

## Materials and methods

This study was conducted as a cross-sectional study analyzing all the malaria parasite (MP)-positive peripheral blood smears of cases reported at the OK Quality Diagnostic Laboratory and Research Center in Peshawar from October 2010 to October 2013. The Ethics Committee of Khyber Medical College/Khyber Teaching Hospital, Peshawar approved this study. OK Quality Laboratory is a welfare diagnostic center that receives patients from across the city, including the main tertiary referral centers. Blood samples of all the patients referred for peripheral blood smear were drawn through venipuncture by professional staff into a 3 mL tube with ethylenediaminetetraacetic acid (EDTA) and were also analyzed for malarial parasites with conventional microscopy. Hemoglobin (Hb), hematocrit (Hct), mean corpuscular volume (MCV), mean corpuscular hemoglobin (MCH), mean corpuscular hemoglobin concentration (MCHC), red blood cell (RBC) count, total leukocyte count (TLC), and platelet count were determined by using a hematology analyzer, HumaReader Plus (HUMAN Diagnostic Worldwide, Wiesbaden, Germany)

All individuals who possessed MP-positive peripheral smears were included in the study and each patient's records were analyzed for age, gender, and species of malarial parasite involved. Various hematological indices were analyzed using the WHO criteria [9]. For hemoglobin, the cut-off criterion indicating anemia was 12 g/dL for females and 13 g/dL for males. Hematocrit was considered to be abnormal at values < 36% for females and < 41% for males. Likewise, RBC count for males was considered normal in the range of 4.2 - 5.8 × 106/cubic millimeter (cmm) and between 3.6 - 5.6 × 106/cmm was considered within normal limits for females. The cut-off values for RBC indices indicating anemia were as follows: MCV < 80 femtoliters (fL), MCH < 27 picograms (pg), and MCHC < 32 grams per deciliter (g/dL). Similarly, platelets < 150 × 103/cmm and TLC values < 4 × 103/cmm were considered abnormal (Table 1).

**Table 1:** WHO Definition for Anemia, Thrombocytopenia, and Leukopenia Based on Various Blood Indices † Cut-off level for males        ‡ Cut-off level for females cmm: cubic millimeter; fL - femtoliters; g/dL: grams per deciliter; pg: picogram

Hematological Variables	World Health Organization (WHO) Criteria [9]
Hematocrit (Hct) (%)	< 41%† & < 36%‡
Hemoglobin (Hb) (g/dL)	< 13 g/dL† & < 12 g/dL‡
Red Blood Cell (RBC) count (× 10⁶/cmm)	< 4.2 × 10⁶/cmm† & < 3.6 × 10⁶/cmm‡
Mean Corpuscular Volume (MCV) (fL)	< 80 fL
Mean Corpuscular Hemoglobin Concentration (MCHC) (g/dL)	< 32 g/dL
Mean Corpuscular Hemoglobin (MCH) (pg)	< 27 pg
Platelet count (× 10^3^/cmm)	< 150 × 10^3^/cmm
Total Leukocyte Count (TLC) (× 10^3^/cmm)	< 4.0 × 10^3^/cmm

All the data were analyzed using the Statistical Package for Social Sciences (SPSS), version 17.0 (IBM SPSS Statistics, Armonk, NY), and the means of different scale variables were analyzed and compared using Student’s t-test and the analysis of variance (ANOVA) test (depending upon the variable groups). Similarly, all the non-parametric variables were analyzed using the Chi-square test of independence (ϰ2). Results were considered statistically significant when the p-value was < 0.05 at 95% level of significance. An extensive literature search was done using the PubMed database and Google Scholar, while references were cited using the Endnote X1 library.

## Results

A total of 136 MP-positive patients were included in the study of which 72 (52.9%) were males and 64 (47.1%) were females with a male to female ratio of 1.12:1. The mean age of patients was 25.8 ± 18.44 years (range: 0.6 - 75 years). Out of total 136 cases, 77 (56.6%) were positive for *P. vivax* malaria, 50 (36.8%) for *P. falciparum* malaria, and nine (6.6%) patients had mixed parasitemia, including both *P. vivax* and *P. falciparum* malarial parasites. However, male to female ratio did not vary significantly across different malarial species, p = 0.84, ϰ2 = 0.958.

**Figure 1:**
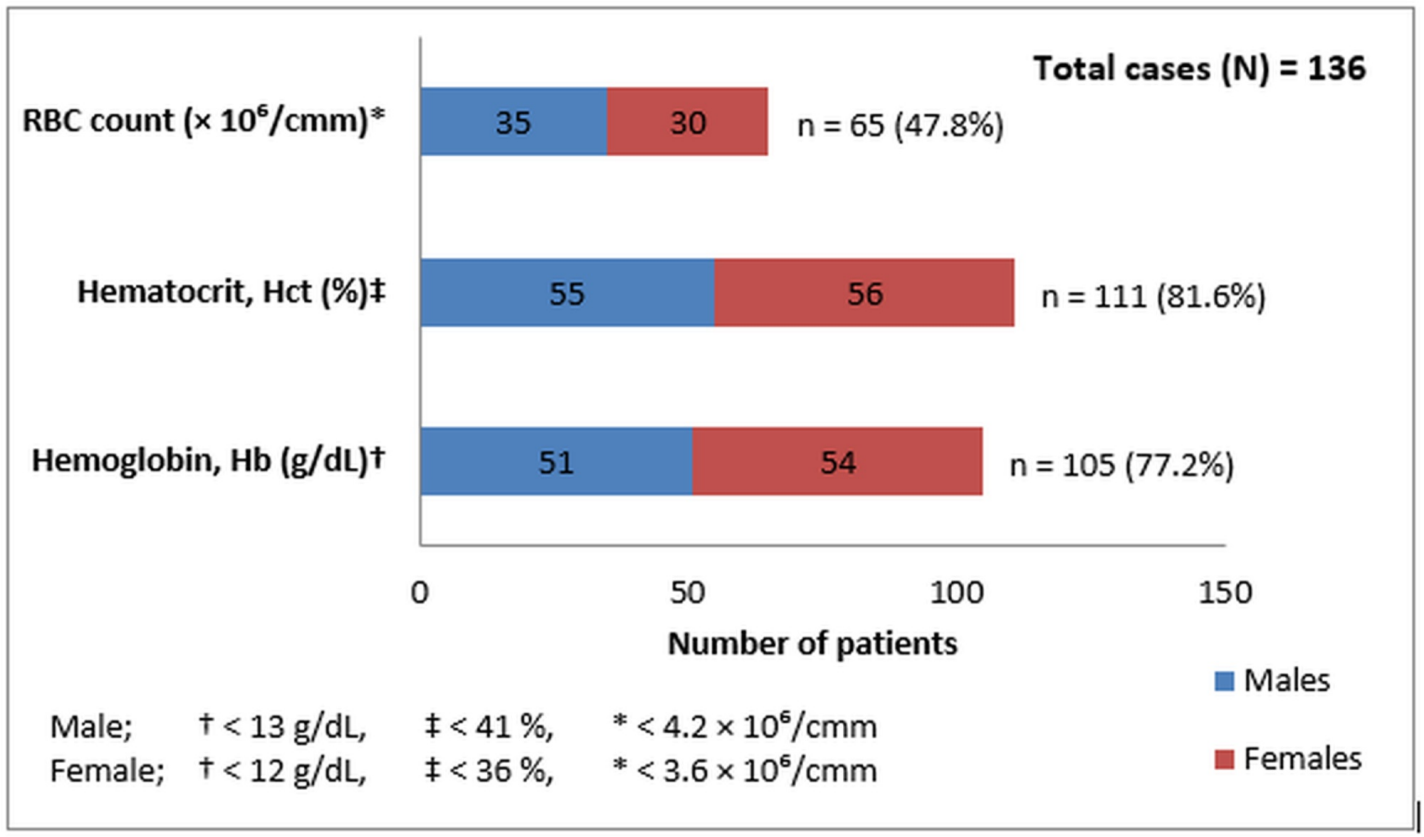
Gender-wise Frequency of Anemic Patients Based on WHO Cut-off Levels for Different Blood Indices WHO: World Health Organization; RBC: red blood cell; cmm: cubic millimeter; g/dL: grams per deciliter; n: number

Mean Hb (10.35 ± 3.4 g/dL), Hct (30.7 ± 9.3%), RBC count (3.9 ± 1.24 × 106/mm^3^), and MCV values (78.2 ± 9.2 fL) were below cut-off limits with regards to WHO criteria [9], thus indicating microcytic hypochromic anemia, whereas means of other variables, like MCH (27.25 ± 7.1 pg), MCHC (33.29 ± 3.4 g/dL), TLC (9.3 ± 7.8 × 103/mm^3^), and platelet count (167 ± 108 × 103/mm^3^), were within normal range. Overall, Hb was low in 77.2% of patients, Hct in 81.6% patients, and MCV was found low in 57.3% cases, again showing that majority of our patients were anemic having a microcytic hypochromic picture. Since cut-off limits for Hb, Hct, and RBC count, based on WHO criteria [9], were different for males and females, Figure 1 further elaborates their gender-wise relative frequencies.

**Figure 2:**
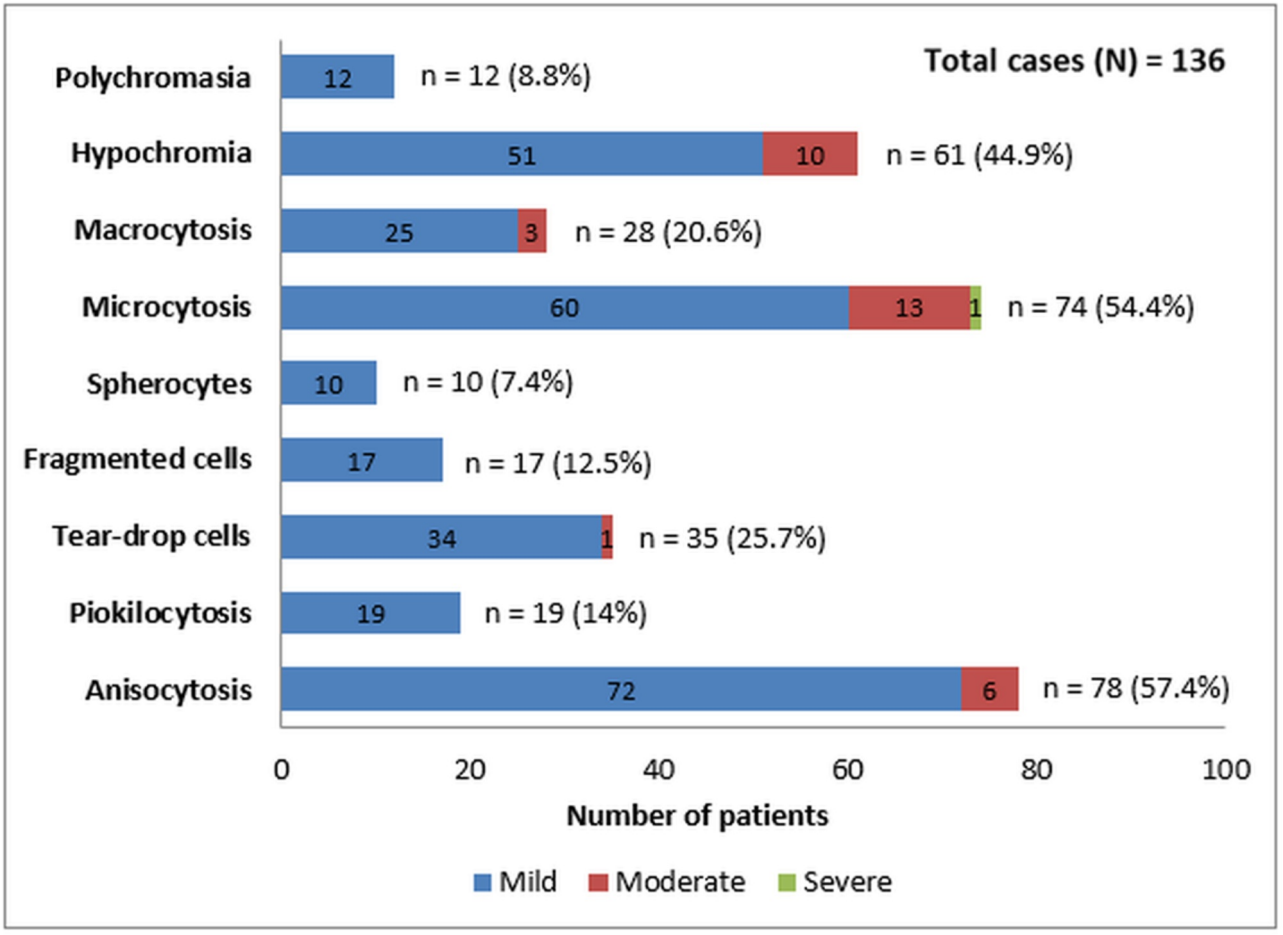
Frequencies of Various Microscopic Findings in Peripheral Blood Smear of MP-positive Patients MP: malaria parasite

Figure 2 shows the prevalence of various other microscopic findings seen on peripheral blood smears of MP-positive patients.

Overall, thrombocytopenia was present in 74 (54.4%) patients and leukopenia was found in 12 (8.9%) patients, while pancytopenia was only present in two (1.5%) patients. In addition, their relative frequencies did not vary significantly with gender, p > 0.05 (Table 2).

**Table 2:** Gender-wise Relative Frequencies of Different Hematological Variables in MP-positive Patients † Cut-off level for males        ‡ Cut-off level for females        * Significant at 95% level of significance MP: malaria parasite; WHO: World Health Organization; P-value: calculated probability; cmm: cubic millimeter; fL: femtoliters; g/dL: grams per deciliter; pg: picogram

Variables	WHO cut-off levels [10]	Males† n = 72	Females ‡ n = 64	Frequency (%)	Chi-square (ϰ^2^)	P – value
Hemoglobin (Hb) (g/dL)	< 13† & < 12‡	51	54	105 (77.2)	3.53	0.06
	≥ 13† & ≥ 12‡	21	10	31 (22.8)		
Hematocrit (Hct) (%)	< 41† & < 36‡	55	56	111 (81.6)	2.79	0.09
	≥ 41† & ≥ 36‡	17	8	25 (18.4)		
Red Blood Cell (RBC) count (× 10⁶/cmm)	< 4.2† & < 3.6‡	35	30	65 (47.8)	0.04	0.84
	≥ 4.2† & ≥ 3.6‡	37	34	71 (52.2)		
​​​​​​​Mean Corpuscular Volume (MCV) (fL)	< 80	41	37	78 (57.3)	0.01	0.92
	≥ 80	31	27	58 (42.7)		
Mean Corpuscular Hemoglobin (MCH) (pg)	< 27	35	37	72 (52.9)	1.15	0.28
	≥ 27	37	27	64 (47.1)		
Mean Corpuscular Hemoglobin Concentration (MCHC) (g/dL)	< 32	29	24	53 (39)	0.1	0.74
	≥ 32	43	40	83 (61)		
Total Leukocyte Count (TLC) (× 10^3^/cmm)	< 4	5	7	12 (8.8)	0.67	0.4
	≥ 4	67	57	124 (91.2)		
Platelet count (× 10^3^/cmm)	< 150	36	38	74 (54.4)	1.2	0.27
	≥ 150	36	26	62 (45.6)		

Interestingly, thrombocytopenia was more common in patients having a *P. vivax* infection (71.4%) as compared to *P. falciparum* (26%) and mixed parasitemia (66.7%) with p = 0.0001. On the other hand, leukopenia was more prevalent in patients with a *P. falciparum* infection (18%) than in *P. vivax* (2.5%) and mixed species infection (11%) with p = 0.01. When analyzed using a Chi-Square test of independence (ϰ2), the frequency of various other hematological variables did not vary significantly in patients with different malarial species (Table 3).

**Table 3:** Relative Frequencies of Various Hematological Indices Among Different Malarial Species † Cut-off level for males        ‡ Cut-off level for females        * Significant at 95% level of significance WHO: World Health Organization; *P*: *Plasmodium*; P-value: calculated probability; N: number; cmm: cubic millimeter; fL: femtoliters; g/dL: grams per deciliter; pg: picogram

Variables	WHO cut-off levels	*P. falciparum* n = 50 (%)	*P. vivax* n = 77 (%)	*P. falciparum* & *P. vivax* n = 9 (%)	Frequency (%)	P – value
Hemoglobin (Hb) (g/dL)	< 12† & < 13‡	40 (80)	57 (74)	8 (88.9)	105 (77.2)	0.5
	≥ 12† & ≥ 13‡	10 (20)	20 (26)	1 (11.1)	31 (22.8)	
Hematocrit (Hct) (%)	< 41† & < 36‡	42 (84)	62 (80.5)	7 (77.8)	111 (81.6)	0.84
	≥ 41† & ≥ 36‡	8 (16)	15 (19.5)	2 (22.2)	25 (18.4)	
Red Blood Cell (RBC) count (× 10⁶/cmm)	< 4.2† & < 3.6‡	27 (54)	33 (42.9)	5 (55.6)	65 (47.8)	0.41
	≥ 4.2† & ≥ 3.6‡	23 (46)	44 (57.1)	4 (44.4)	71 (52.2)	
Mean Corpuscular Volume (MCV) (fL)	< 80	34 (68)	39 (50.6)	5 (55.6)	78 (57.4)	0.15
	≥ 80	16 (32)	38 (49.4)	4 (44.4)	58 (42.6)	
Mean Corpuscular Hemoglobin (MCH) (pg)	< 27	25 (50)	42 (54.5)	5 (55.6)	72 (52.9)	0.86
	≥ 27	25 (50)	35 (45.5)	4 (44.4)	64 (47.1)	
Mean Corpuscular Hemoglobin Concentration (MCHC) (g/dL)	< 32	16 (32)	34 (44.2)	3 (33.3)	53 (39)	0.36
	≥ 32	34 (68)	43 (55.8)	6 (66.7)	83 (61)	
Total Leukocyte Count (TLC) (× 10^3^/cmm)	< 4	9 (18)	2 (2.6)	1 (11.1)	12 (8.8)	0.01*
	≥ 4	41 (82)	75 (97.4)	8 (88.9)	124 (91.2)	
Platelet count (× 10^3^/cmm)	< 150	13 (26)	55 (71.4)	6 (66.7)	74 (54.4)	< 0.001*
	≥ 150	37 (74)	22 (28.6)	3 (33.3)	62 (45.6)	

Using hematological indices as scale variables mean hemoglobin concentration was 9.1 g/dL in subjects with *P. falciparum* malaria, 10.7 g/dL in patients with *P. vivax,* and 9.86 g/dL in cases having a mixed infection with *P. Falciparum* and *P. vivax* parasites. However, the ANOVA test did not reveal any statistically significant variation in mean hemoglobin concentration across different malarial species, p = 0.39. The lowest hemoglobin concentration was 2.7 g/dL in *P. falciparum* infection and 5.4 g/dL in *P. vivax* parasitemia. The mean platelet count in *P. vivax* malaria was 135.8 ± 89.4 × 103/cmm as compared to *P. falciparum* and mixed species infection, where the mean platelet count was 222 ± 118.7 × 103/cmm and 141.8 ± 70.5 × 103/cmm, respectively. The ANOVA test showed the difference to be statistically significant with F = 11.5, p < 0.0001. Likewise, the mean values of the Hct, MCV, and MCHC showed a statistically significant variation when compared across different malarial species, p < 0.05 (Table 4).

**Table 4:** Mean Values of Various Hematological Indices Across Different Malarial Species * Significant at 95% level of significance P: *Plasmodium*; P-value: calculated probability; SD: standard deviation; cmm: cubic millimeter; fL: femtoliters; g/dL: grams per deciliter; pg: picogram

VARIABLES	P. falciparum Mean ± SD	P. vivax Mean ± SD	P. falciparum & P. vivax Mean ± SD	ANOVA (F)	P – value
Hematocrit (Hct) (%)	27.8 ± 10.74	32.7 ± 7.17	30.6 ± 13.0	4.36	0.01*
Hemoglobin (Hb) (g/dL)	9.9 ± 4.48	10.7 ± 2.36	9.86 ± 3.81	0.94	0.39
Mean Corpuscular Volume (MCV) (fL)	76.52 ± 9.9	79.94 ± 6.43	73.7 ± 19.0	3.4	0.03*
Red Blood Cell (RBC) count (× 10⁶/cmm)	3.65 ± 1.51	4.08 ± 0.92	3.82 ± 1.76	1.87	0.15
Mean Corpuscular Hemoglobin Cenctration (MCHC) (g/dL)	34.32 ± 3.6	32.68 ± 3.2	32.7± 3.3	3.7	0.02*
Mean Corpuscular Hemoglobin (MCH) (pg)	27.93 ± 8.6	26.34 ± 3.26	31.25 ± 16.37	2.3	0.1
Platelet count (× 10^3^/cmm)	222 ± 118.7	135.8 ± 89.4	141.8 ± 70.5	11.5	< 0.0001*
Total Leukocyte Count (TLC) (× 10^3^/cmm)	10.4 ± 11.4	8.7 ± 4.5	8.12 ± 4.3	0.86	0.4

Analyzing various hematological indices as categorical variables and using cut-off levels in accordance with WHO criteria of anemia [9], the Chi-square test (ϰ2) revealed no gender-wise statistically significant variation in their relative frequencies, p > 0.05 (Table 5). In contrast, when analyzing these indices as scale variables, the mean Hct was found to be 32.7 ± 9.2% in males and 28.5 ± 8.9% in females. Using the Student’s T-test, such variation was found to be statistically significant, p = 0.007. However, other variables did not show any significant gender-wise variation in their relative frequencies when analyzed as a scale variable using the Student’s T-test (Table 5).

**Table 5:** Means of Various Hematological Variables in MP-positive Males and Females * Significant at 95% level of significance MP: malaria parasite; SD: standard deviation; P-value: calculated probability; cmm: cubic millimeter; fL: femtoliters; g/dL: grams per deciliter; pg: picogram

VARIABLES	Males Mean ± SD	Females Mean ± SD	Student’s T-test	P – value
Hemoglobin (Hb) (g/dL)	10.8 ± 3.13	9.7 ± 3.6	1.91	0.058
Hematocrit (Hct) (%)	32.7 ± 9.2	28.5 ± 8.9	2.7	0.008*
Red Blood Cell (RBC) count (× 10⁶/cmm)	4.09 ± 1.08	3.70 ± 1.37	1.85	0.06
Mean Corpuscular Volume (MCV) (fL)	77.3 ± 11.16	79.3 ± 6.19	1.27	0.20
Mean Corpuscular Hemoglobin (MCH) (pg)	27.84 ± 9.12	26.5 ± 3.65	1.09	0.27
Mean Corpuscular Hemoglobin Concentration (MCHC) (g/dL)	33.16 ± 3.66	33.43 ± 3.19	0.46	0.64
Total Leukocyte (TLC) (× 10^3^/cmm)	9.2 ± 9.33	9.47 ± 5.6	0.20	0.83
Platelet count (× 10^3^/cmm)	180.1 ± 107.7	154.2 ± 107.1	1.40	0.16

## Discussion

Malaria typically affects blood indices in various ways with anemia and thrombocytopenia being the frequent associated hematological outcomes. Studies have reported thrombocytopenia as a sensitive marker for a malaria diagnosis in the presence of acute febrile illness, having a sensitivity of 60%, a specificity of 88% [10], and a positive and negative predictive value of 86% and 100%, respectively [11]. Although the exact mechanism of thrombocytopenia in malaria is still a topic of extensive worldwide research, studies have considered immunoglobulin G (IgG)-mediated platelet destruction [12], sequestration in the spleen, oxidative stress, and abnormalities in platelets’ structure caused by the invasion of the parasite as possible explanations [13-14]. Researchers have also suggested thrombocytopenia as a result of consumption by disseminated intravascular coagulation (DIC) and peripheral platelet destruction induced by P. falciparum, although the latter mechanism has not been systematically evaluated in *P. vivax* malaria [8]. Similarly, studies have considered the peripheral destruction of RBCs, ineffective hematopoiesis, and sequestration in the spleen as possible causes of malaria-induced anemia [8]. Likewise, the role of TNF and IL-10 have been studied in the development of *P. falciparum*-induced anemia; however, such a role has not been observed in the development of malaria-induced thrombocytopenia [15].

Mild to severe thrombocytopenia should alert the possibility of a malarial infection, with thrombocytopenia being a frequent complication of *P. vivax* infection. Kochar et al. has shown the association of thrombocytopenia with *P. vivax* monoinfection as more significant when compared to thrombocytopenia in *P. falciparum* monoinfection (odds ratio (OR) = 2.335 (95% CI; 1.72 – 3.16), p < 0.0001) [13]. In the present study overall, 54.4% of our patients suffering from malaria showed thrombocytopenia. Out of the total 77 cases having a *P. vivax* infection, 71.4% patients had *P. vivax*-associated thrombocytopenia (Table 3). These figures were in good agreement with studies done by other investigators showing P. vivax-associated thrombocytopenia in 63% by Khan et al. [16], 82% by Srivastava et al. [17], and 93.3% by George and Alexander [18]. On the contrary, studies, such as Rodriguez-Monrale et al. [19] and Gonzalez et al. [20], have shown *P. vivax*-associated thrombocytopenia in 58.9% and 55.9% cases, respectively.

According to our study, only 26% of cases with *P. falciparum* infection had associated thrombocytopenia. Although this was in good agreement with studies like Casals-Pascual et al. [21] and Tan et al. [22] showing *P. falciparum*-related thrombocytopenia in 34.4% and 34% cases, respectively, others have reported much higher frequencies. This includes 51.8% by Noronha [23] and 85% by Prasad et al. [24].

In our study, the mean Hb in females (9.7 ± 3.6 g/dL) was lower than that in males (10.8 ± 3.13 g/dL) with p = 0.058 (Table 5). These results were in good agreement with studies from Columbia and Uganda [25-26], showing a mean Hb in females lower than those of males in malaria patients. According to WHO criteria of anemia [9], 105 (77.2%) of our patients were anemic with regards to Hb values (Figure 1). Similarly, a study from Uganda [26] had shown 76.8% prevalence of malaria parasitemia among severely anemic hospitalized patients in malaria-endemic regions. In our study, out of 105 anemic patients, 57/105 (54.3%) had a *P. vivax* infection, 40/105 (38.1%) had *P. falciparum* while 8/105 (7.6%) had mixed parasitemia. However, out of nine patients with mixed parasitemia, eight (88.9%) had anemia, while 40/50 (80%) of *P. falciparum* and 57/77 (74%) of *P. vivax* patients had anemia according to WHO criteria (Table 3). Again, these results were comparable to a study from Gabon [25], where the prevalence of anemia was reported between 87.6 - 90.7% among patients having *P. falciparum* parasitemia.

In addition to having abnormalities in RBC and platelet counts, studies have reported low to normal total leukocyte counts in malaria [27-28], although none of these abnormalities are specific to malaria [29]. Likewise, Sharma et al. [29] have reported high sensitivity and positive predictive value (PPV) for a malaria diagnosis in patients with abnormal scattergram. However, in our study, only 12/136 (8.9%) patients showed leukopenia, which was contrary to results shown by Shaikh et al. [1] who reported leukopenia in 35.4% of malaria patients. In our study, leukopenia was more common in patients suffering from *P. falciparum* infection (18%) than from *P. vivax* (2.5%) and mixed infections (11%). This was again in contrast to studies, like McKenzie et al. [28], that showed white blood cell (WBC) counts in *P. falciparum*-infected patients were lower than those having an infection with *P. vivax*, which in turn, were lower than those in uninfected patients.

## Conclusions

Malaria is frequently associated with anemia (77.2%), thrombocytopenia (54.4%), and leukopenia (8.8%) of cases. In patients with anemia, 54.3% had *P. vivax* parasitemia, whereas 38.1% had *P. falciparum* infection. Thrombocytopenia was associated with *P. vivax* infection in 71.4% cases, whereas 26% had *P. falciparum* infection. On contrary, leukopenia was more prevalent in *P. falciparum* patients (18%), followed by *P. vivax* (2.6%) and mixed parasitemia (11.1%).
